# Localized light chain amyloidosis: A self-limited plasmacytic B-cell lymphoproliferative disorder

**DOI:** 10.3389/fonc.2022.1002253

**Published:** 2022-11-15

**Authors:** José C. Martínez, Eben I. Lichtman

**Affiliations:** ^1^ Division of Hematology, Department of Medicine, School of Medicine, University of North Carolina at Chapel Hill, Chapel Hill, NC, United States; ^2^ Lineberger Comprehensive Cancer Center, University of North Carolina at Chapel Hill, Chapel Hill, NC, United States

**Keywords:** plasmacytoma, amyloid, immunoglobulin light chain, amyloidosis, giant cells, lymphoma, plasma cell disorder

## Abstract

Immunoglobulin light chain amyloidosis can be either systemic or localized. Although these conditions share a similar name, they are strikingly different. Localized light chain amyloidosis has been challenging to characterize due to its lower incidence and highly heterogeneous clinical presentation. Here, we review the emerging literature, emphasizing recent reports on large cohorts of patients with localized amyloidosis, and provide insights into this condition’s pathology and natural history. We find that patients with localized amyloidosis have an excellent prognosis with overall survival similar to that of the general population. Furthermore, the risk of progression to systemic disease is low and likely represents initial mischaracterization as localized disease. Therefore, we argue for the incorporation of more sensitive techniques to rule out systemic disease at diagnosis. Despite increasing mechanistic understanding of this condition, much remains to be discovered regarding the cellular clonal evolution and the molecular processes that give rise to localized amyloid formation. While localized surgical resection of symptomatic disease is typically the treatment of choice, the presentation of this disease across the spectrum of plasmacytic B-cell lymphoproliferative disorders, and the frequent lack of an identifiable neoplastic clone, can make therapy selection a challenge in the uncommon situation that systemic chemotherapy is required.

## Introduction

The famous German physician-scientist Rudolph Virchow adopted the term amyloid—referring to “starch-like” or “cellulose-like” from Greek—in 1854 to describe macroscopic tissue deposits that were stained with iodine (1). He coined this term based on the wrong assumption that amyloid was composed of polysaccharides. Technical advances in the last century led to the discovery that amyloid consists of proteins with a fibrillar structure (1, 2). All amyloid fibrils share a cross beta-sheet structure (3) and bind to Congo red dye, giving it the characteristic birefringence appearance under polarized light microscopy (4). These insoluble protein aggregates are the result of protein misfolding (5). Researchers have identified an astoundingly diverse (36 to date) repertoire of precursor proteins that form amyloid (6).

Amyloidosis refers to the heterogeneous group of conditions that result in amyloid fibril production and tissue deposition (7). It is classified by the precursor amyloidogenic protein, and it can be further distinguished by whether there is systemic vs. localized organ involvement. Systemic immunoglobulin light chain (AL) amyloidosis is the most prevalent form of amyloidosis (8). In this condition, bone marrow-derived circulating immunoglobulin-free light chains produce amyloid fibrils (9). Almost every organ can be affected by amyloid deposition except the brain (5). However, this condition affects the heart and kidneys most frequently. Systemic AL amyloidosis has high morbidity and mortality, especially if there is advanced organ failure at diagnosis (10, 11). Therefore, an early diagnosis is essential to halt the progression of end-organ damage. Hence, it is imperative to distinguish systemic AL amyloidosis from localized light chain (AL_L_) amyloidosis.

AL_L_ amyloidosis results from confined amyloid deposition in a single organ. It is a rare entity representing 7-12% of all amyloidosis (12–14). In localized AL_L_ amyloidosis, plasmacytic B-cells reside in the affected organs, producing immunoglobulin-free light chains locally (15, 16). In contrast to systemic AL amyloidosis, there are often no circulating monoclonal immunoglobulin light chains (17). Any organ can be involved in AL_L_ amyloidosis, including the nervous system (18, 19). However, the larynx, trachea, lung, skin, and urinary tract are the most frequently affected (12–14). AL_L_ amyloidosis has an excellent prognosis (12–14).

The origins of AL_L_ amyloidosis remain a mystery. Until recently, most of our knowledge of this condition came from case reports and small case series. However, emerging literature reporting on large patient cohorts is helping us to better understand this disease. We will review this new evidence and provide suggestions for future research directions.

## Disease pathology

In the 1970s and 1980s, improvements in biochemical techniques led to the identification of immunoglobulin light chains in amyloid fibrils derived from localized tumors (20–22). However, the source of amyloidogenic immunoglobulin light chains was not immediately apparent. This was difficult to assess due to 1) the sparse presence of plasmacytic B-cells in the affected organ and 2) technical limitations in establishing clonality and biochemical characterization of amyloid fibril composition. Nevertheless, later studies confirmed that in some cases, a localized clonal plasmacytic B-cell population was the source of immunoglobulin light chain production (16, 18, 23–25). More recently, in a large cohort of patients with AL_L_ amyloidosis, a lymphoplasmacytic infiltrate could be identified in 49% of cases and clonality established in 30% of cases (14). Further characterization of the lymphoplasmacytic infiltrates associated with localized amyloidosis reveals that B-cells are often present in conjunction with plasma cells (26). However, it remains unclear whether this B cell component represents a neoplastic clone. Additional research is needed to better classify the lymphoproliferation associated with AL amyloidosis.

Early studies reported similar kappa to lambda ratios in the immunoglobulin light chain composition in localized amyloid fibrils (15). This differs from systemic AL amyloidosis, where lambda-free light chains predominate in a 3:1 ratio (27). Reports from large AL_L_ amyloidosis patient cohorts find disparate kappa to lambda ratios of 3:1 (12), 1.4:1 (13), and 1:3 (14). A significant limitation of early studies is the small number of cases that included amyloid typing. Basset et al. (14) carried the most comprehensive typing by including immunohistochemistry from 246 patient samples. This study revealed a tissue-specific bias in amyloid light chain composition where lambda predominates in the urinary tract, GI, skin, and CNS, and kappa predominates in the lymph nodes (14). However, they relied on immunohistochemistry for all their analysis and only included eight patients with lymphoid AL_L_. Although Kourelis et al. (13) reported typing on a lower number of cases, they included the highest number of samples analyzed with mass spectrometry. Overall, these findings suggest that lambda light chains might also be the predominant isotype in AL_L_ amyloidosis. Future studies utilizing more sensitive amyloid typing techniques such as mass spectrometry might clarify this question.

**Figure 1:**
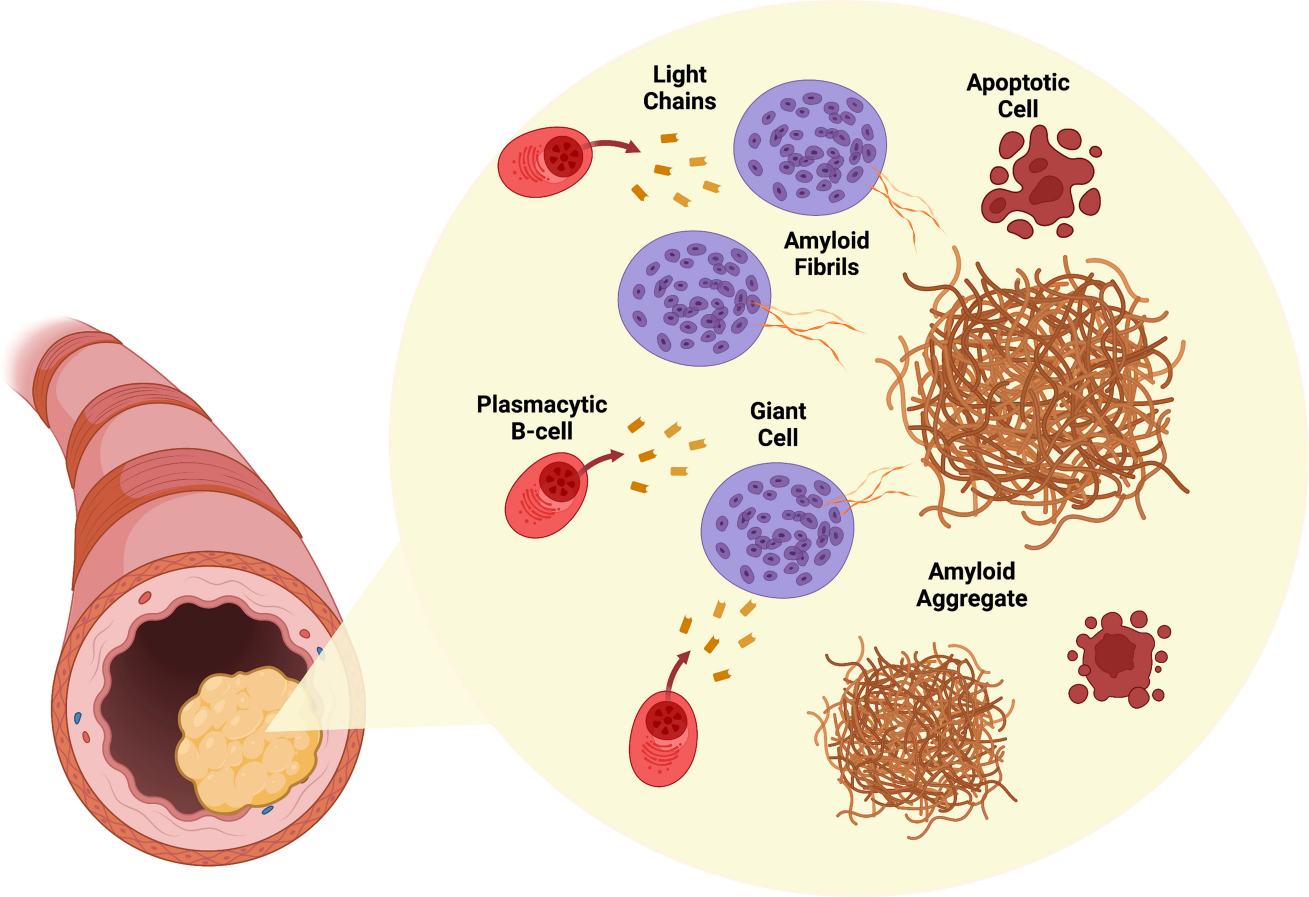
Pathogenesis of AL_L_ amyloidosis. Infiltrating plasmacytic B-cells locally produce amyloidogenic light chains. Surrounding giant cells process these light chains into amyloid fibrils. Accumulation of amyloid fibrils in the involved organ (larynx shown) results in tumor growth. Cytotoxic amyloid fibrils are toxic to plasmacytic B-cells. Created with BioRender.com.

How, where, and why locally produced immunoglobulin light chains become amyloid fibrils remains unclear. Interestingly, amyloidogenic variable regions in the light chains (VL) undergo immune-driven selection (28). Mutations in the VL resulting in destabilizing amino acid replacements are linked to the amyloidogenic process (29). An interesting finding is that multinucleated giant cells are exclusively found in amyloid deposits in AL_L_ amyloidosis and are notably absent in systemic AL (30). Given the close deposition of amyloid fibrils and specific orientation around these giant cells as seen under electron microscopy, it has been proposed that these cells could play a role in amyloid fibril generation (15, 30). Based on these findings, Westermark further hypothesizes a pathogenic mechanism by which local clonal plasmacytic B-cells produce amyloidogenic proteins that are processed into amyloid fibrils in these giant cells leading to toxic amyloid fibril deposition and death of the original plasmacytic B-cell clones (15). **Figure 1** shows a representative schema of this proposed mechanism.

## Diagnosis

AL_L_ amyloidosis presents in clinically diverse ways due to the many distinct organs it can involve. Often, a tissue biopsy with histologic confirmation of amyloidosis is available at diagnosis. Thus, the diagnostic workup generally involves amyloid typing and ruling out a systemic B-cell lymphoproliferative disorder (most commonly systemic AL amyloidosis, multiple myeloma, and B-cell lymphoma). Therefore, testing should be performed to a) confirm immunoglobulin-derived amyloid fibrils with typing, ideally *via* liquid chromatography-tandem mass spectrometry (LC-MS/MS) (31), b) evaluate for evidence of a clonal plasma cell or B-cell population, c) rule out systemic organ involvement with AL amyloidosis, and d) characterize potential manifestations of localized organ involvement (32–34).

For situations in which AL_L_ is suspected in a commonly involved site such as urothelial, laryngeal, pharyngeal, or tracheobronchial, initial hematologic evaluation for a monoclonal protein is typically sufficient to rule out systemic AL amyloidosis (13). Evaluation should include complete hematologic staging incorporating fat pad and bone marrow biopsies if there is a higher pre-test probability of systemic AL amyloidosis. For every patient, evaluation for evidence of potential systemic amyloidogenic free light chains should include a serum and 24-hour urine protein electrophoresis with immunofixation and serum-free light chains (35, 36). A fat pad biopsy can be included in cases where the involved organ is classic for AL_L_ amyloidosis (e.g., urothelial, laryngeal, or tracheobronchial) (13). A bone marrow biopsy should be performed for those with atypical organ involvement, such as in the gastrointestinal tract. In cases where the lungs or lymph nodes are involved, imaging with PET-CT should be considered, depending on clinical history, to evaluate for a systemic B-cell lymphoma. A helpful characteristic of amyloid fibrils is that it binds to serum amyloid P component (SAP) (37). This provides a target for imaging amyloid deposition *in-vivo* (38, 39) and therapy (40). At certain amyloidosis referral centers and where available, SAP scintigraphy can also be used to assess for systemic organ involvement with high sensitivity and specificity (41).

In addition to a thorough clinical evaluation, specific markers of end-organ damage can be obtained, including N-terminal pro-brain natriuretic peptide (NT-proBNP) or brain natriuretic peptide (BNP), alkaline phosphatase, and 24-hour urine protein quantification or spot urine protein/creatinine ratio to assess for cardiac, hepatic, and renal involvement, respectively. Cardiac organ involvement is of particular concern as it is associated with high mortality. Nuclear imaging with ^99^mTc- labeled 3,3-diphosphono-1,2-propanodicarboxylic acid (DPD) or ^99^mTc- labeled pyrophosphate (PYP) bone tracer scintigraphy is helpful to evaluate suspected cardiac transthyretin (ATTR) amyloidosis in the absence of a detectable monoclonal protein, but is not relevant to the evaluation of suspected AL_L_ and cannot be used to reliably distinguish cardiac ATTR from AL amyloidosis in the presence of a detectable monoclonal protein (42, 43).

Mass spectrometry offers significant advantages in the diagnosis of amyloidosis as well as in the detection and monitoring of monoclonal free light chains. Liquid chromatography-tandem mass spectrometry (LC-MS/MS) is an unbiased, more efficient method of amyloid typing compared to classical immunohistochemistry techniques (31). As such, it should be the gold standard approach to amyloid typing. Serum-free light chain matrix-assisted laser desorption/ionization-time of flight (MALDI-TOF) mass spectrometry (44) is a novel method that has increased detection sensitivity for diagnosing and monitoring amyloidogenic free light chains (45, 46) and recent guidelines have recommended incorporating this method has into routine clinical practice (47). Therefore, continued effort should be placed on incorporating and standardizing these techniques for clinical practice.

## Epidemiology

AL_L_ amyloidosis is rare and has been mainly described in case reports or small case series until recently. However, three retrospective studies on large cohorts of patients with AL_L_ amyloidosis provide new insights into the epidemiology and natural history of the disease. These studies report on data collected at the UK National Amyloidosis Centre (12), Mayo Clinic (13), and Heidelberg Amyloidosis Center (14). AL_L_ amyloidosis represented 7-12% of all cases of amyloidosis at these centers. The median age at diagnosis was 58-59.5 years. Both sexes were equally affected.

A monoclonal protein component was present in 13% (12), 7% (13), and 22% (14) of cases. This is higher than expected in the general population, with a prevalence of around 4% in this age group (48). Moreover, a concomitant lymphoma either in the form of marginal zone lymphoma (MZL) or mucosa associated lymphoid tissue (MALT) tumor was seen in 1-4% of cases in these cohorts (12–14). In one case series, the lymphomatous infiltrate was confirmed to be present at the site of amyloid deposition in 5 out of 7 (71%) patients (14). There is also a higher incidence of co-occurring autoimmune diseases, with rates of 11% (12), 7% (13), and 21% (14). Sjögren syndrome is the most common co-occurring autoimmune disorder, followed by autoimmune thyroiditis and rheumatoid arthritis. As previously reviewed, the lungs and the skin were the most frequently involved sites in patients with AL_L_ amyloidosis and Sjögren syndrome (49). Interestingly, AL_L_ involvement in these sites often co-occurs with MALT lymphomas as reported in 10 of 52 patients (19%) with lung AL_L_, and 6 of 53 (11%) patients with skin AL_L_ (13). This is consistent with prior reports describing an association between MALT lymphomas and Sjögren syndrome with localized amyloid production (50, 51).

## Clinical presentation

The site of amyloid involvement determines the clinical symptoms at presentation. The larynx, trachea, lung, skin, and urinary tract are the most frequently involved organs (12–14). Multifocal single organ involvement without evidence of systemic disease is reported in around 44% of cases (14). Multifocal involvement is more common in the skin, gastrointestinal tract (12), and lungs (14). At initial diagnosis, symptoms secondary to localized amyloid deposition are present in 66-72% of patients (13, 14). The median time from symptom onset to diagnosis is seven months (12, 13). Patients with laryngeal (98%) and urinary tract (88%-95%) involvement at diagnosis (13, 14) are more frequently symptomatic. On the contrary, patients with lung (13, 14), GI tract (13), and skin (14) involvement have a lower frequency of symptoms.

Localized amyloid deposits may grow into tumor-like lesions and produce site-specific symptoms due to their mass effect. Common symptoms include hoarseness and dyspnea secondary to laryngeal obstruction, hematuria, and recurrent urinary tract infections secondary to the bladder or urethral involvement. **Table 1** summarizes the clinical presentations and outcomes of patients with AL_L_ amyloidosis.

**Table 1:** Presentation and outcomes of patients with localized amyloidosis.

	Mahmood et al., 12	Kourelis et al., 13	Basset et al., 14
	*N* = 606	*N* = 413	*N* = 293
Age: median (range)	59.5 (48–87)	59 (13-91)	58 (18-83)
Sex	51% male 49% female	48% male 52% female	49% male 51% female
Monoclonal Protein	76 (13%)	27 (7%)	63 (22%)
Autoimmune Disease	67 (11%)	28 (7%)	61 (21%)
Light chain subtype			
Lambda	67 of 501 (13%)	67 (16%)	217 (74%)
Kappa	24 of 501 (5%)	93 (22%)	76 (26%)
Undetermined	410 of 501 (82%)	243 (59%)	0
Involved System			
Respiratory	174 (29%)	149 (36%)	157 (54%)
Urinary	115 (19%)	85 (21%)	37 (13%)
Skin	94 (16%)	53 (13%)	31 (11%)
Gastrointestinal	72 (12%)	62 (15%)	35 (12%)
Eye-related	70 (12%)	35 (9%)	12 (4%)
Lymphatic	31 (5%)	5 (1%)	8 (3%)
Other	50 (8%)	24 (6%)	13 (4%)
Symptomatic	Not reported	297 (72%)	194 (66%)
Required treatment	270 of 527 (51%)	287 (70%)	163 (56%)
Treatment Modality			
Surgical excision	228 of 270 (84%)	252 of 287 (88%)	135 of 163 (83%)
Radiotherapy	4 of 270 (2%)	25 of 287 (9%)	5 of 163 (3%)
Other	38 of 270 (14%)	10 of 287 (4%)	23 of 163 (14%)
Multiple treatments	122 (20%)	61 (15%)	Not reported
5-year overall survival	91%	92%	94%

## Management

Half to three-quarters of patients with AL_L_ amyloidosis require treatment (12–14). The primary indication for treatment is symptom control. A little over 70% of patients experience symptomatic improvement after initial treatment (13, 14). The extent (focal vs. multifocal), resectability, and clonality of the disease, guide the choice of treatment modality. Surgical excision is the most common first-line therapy, with rates of 84% (12), 61% (13), and 83% (14) in respective case series. This is the only curative treatment modality as it can remove both amyloid deposits and amyloidogenic B-cells. Other treatment modalities are aimed at ameliorating amyloid fibril production by targeting the amyloid-producing B-cells. These treatments include radiotherapy, systemic or local steroids injections, and rarely chemotherapy. Radiotherapy can effectively stabilize refractory and locally advanced disease that is not amenable to surgery (12–14, 52, 53). However, it does not address already established amyloid deposits. Other, less commonly used but reported treatments include local application of dimethyl-sulfoxide and colchicine (12, 13, 54, 55). Systemic chemotherapy is used in cases where local approaches would be ineffective (e.g multifocal disease in difficult to radiate areas) or when a secondary lymphoid malignancy is present, which overall represents <1% of patients (12–14). The overlap and spectrum of clonal B-cell differentiation complicates decision making regarding type of systemic chemotherapy. A specific B-cell clone is identified half of the time and often both B-cells and plasma cells are present in biopsy samples thus making it difficult to decide whether to pursue B-cell directed or plasma-cell directed therapy.

## Outcomes and prognosis

Patients with AL_L_ amyloidosis have an excellent prognosis with median overall survival (OS) comparable to that of the general population (12–14). Median OS at 5 and 10 years were reported to be 90.6% and 80.3% (12), 92% and 78% (13), and 94% and 92% (14), among these large case series. Patients with lung AL_L_ had a decreased OS compared to other groups (13, 14). However, these patients are a decade older at diagnosis and more likely to have co-existing autoimmune conditions and smoke (13, 14). Only age is associated with an increased risk of death (hazard ratio, 1.1; 95% CI, 1.08-1.14) when multivariable analysis is performed (13). AL_L_ amyloidosis was directly linked to the cause of death in less than 1% of cases (12–14). However, the cause of death was not known in many cases, and the median follow-up in these studies was 4-6 years.

Clinicians worry that AL_L_ amyloidosis will progress into systemic disease. However, multiple studies demonstrate very low or no risk of progression to systemic AL amyloidosis (12–14, 17, 53). Only two studies report progression to systemic disease with incidences of 7 out of 606 patients (12) and 3 out of 293 patients (14) respectively. Notably, most of these patients had either lymph node or lung involvement, and most had a detectable monoclonal protein component. Lymphadenopathy can be a site of early systemic AL amyloidosis, which carries a risk of progression to additional organ involvement over time (56). Therefore, patients with suspected AL_L_ amyloidosis of the lymph nodes or lungs should undergo stricter evaluation and monitoring to rule out systemic AL amyloidosis.

Approximately one in five patients will require more than one intervention or have a recurrence after the first line of treatment (12–14). The disease recurs at a median time of 41 months (13) and 43 months (14) respectively. A common site of disease recurrence is the larynx (12, 14, 53). Local progression is also relatively common and affects up to one-third of patients (14). Rates of 5-year progression-free survival are estimated at 77% (13) and 62% (14). Patients with urothelial AL_L_ have a lower rate of 5-year progression-free survival compared to the entire cohort (67% vs. 82%) (13).

## Discussion and future directions

This review finds substantial evidence to support the hypothesis that AL_L_ amyloidosis is a self-limited lymphoplasmacytic disorder. Of note, self-limited in this context refers to the low likelihood of localized amyloidosis progressing to systemic disease. However, local progression can still be associated with significant morbidity and require treatment. Overall, AL_L_ amyloidosis has an excellent prognosis with an overall survival similar to that of the general population. It is debated whether progression to systemic disease is a true feature of AL_L_ amyloidosis or whether it represents mischaracterization of original cases of systemic AL amyloidosis. Generally, these conditions have distinct patterns of organ involvement. However, disease initially localized to specific organs such as the lymph nodes and lungs appears to have a higher risk of “progression” to systemic involvement. Therefore, such cases should be closely monitored and thoroughly worked up for evidence of a plasma cell clone.

The clinical presentation of AL_L_ amyloidosis is highly heterogeneous and determined by the site of involvement. Patients with laryngeal and urinary tract involvement are most symptomatic at diagnosis. In most cases, there is a symptomatic improvement with localized treatment consisting of either surgical excision or involved-site radiotherapy. Unfortunately, disease recurrence, as well as local progression, are common. Overall, it appears that radiotherapy is underutilized in the first line. Future studies assessing the effectiveness of radiotherapy vs. surgical interventions in the first lines are warranted. Other less common interventions, such as intravesical DMSO instillation (for bladder AL_L_), colchicine injections, and steroids, have no evidence of efficacy. Systemic chemotherapy is primarily utilized in cases where there is a concomitant lymphoma. It would be interesting to assess whether clone-directed chemotherapy has a role in exceptional cases where surgical excision or radiotherapy is not feasible, and treatment is required.

The pathogenesis of AL_L_ amyloidosis remains poorly understood. Despite significant technical advances, it is still challenging to characterize the plasmacytic B-cells present in these tumors. A lymphoplasmacytic infiltrate is not detected in more than half of the cases. Clonality is established with even lower resolution. The application of novel techniques, such as single-cell RNA sequencing combined with laser capture microdissection and *in-situ* RNA sequencing, to the study of cancer biology is promising (57). If applied in this context, it could provide insights into the origins and mechanisms underlying amyloidogenic plasmacytic B-cell selection. As noted, there is a higher prevalence of co-occurring lymphoproliferative disorders such as monoclonal gammopathy, MALT, and MZL lymphomas, with autoimmune conditions, such as Sjögren’s syndrome, in patients with AL_L_ amyloidosis. This finding, together with evidence that most involved sites are mucosal, skin, and lungs, suggests that chronic antigen exposure or autoimmunity plays a role in the development of clonality (12). However, the inciting antigen exposure or immune response that signals plasmacytic B-cell infiltration, activation, and selection, is unknown and requires additional research.

## Author contributions

JM reviewed the literature and wrote the review article. EL conceived and guided the study, wrote and critically reviewed the article for important intellectual content. All authors approved the final version for submission. All authors contributed to the article and approved the submitted version.

## Conflict of interest

The authors declare that the research was conducted in the absence of any commercial or financial relationships that could be construed as a potential conflict of interest.

## Publisher’s note

All claims expressed in this article are solely those of the authors and do not necessarily represent those of their affiliated organizations, or those of the publisher, the editors and the reviewers. Any product that may be evaluated in this article, or claim that may be made by its manufacturer, is not guaranteed or endorsed by the publisher.
